# Robotic Adrenalectomy and Clevidipine: A New Frontier in Pheochromocytoma Management Preliminary Study

**DOI:** 10.3390/jcm14041103

**Published:** 2025-02-09

**Authors:** Nunzia Cinzia Paladino, Carole Guérin, Anderson Loundou, Nancy Domato, Cedric Atondeh, David Taïeb, Frédéric Sebag

**Affiliations:** 1 Department of General and Endocrine Surgery, Conception University Hospital, Aix-Marseille University, 147, Boulevard Baille, 13005 Marseille, France; 2 Support Unit for Clinical Research and Economic Evaluation, Department of Research and Innovation, Aix-Marseille University, 264, Rue Saint Pierre, 13385 Marseille, France; 3 Department of Nuclear Medicine, Timone University Hospital, Aix-Marseille University, 147, Rue Saint Pierre, 13005 Marseille, France

**Keywords:** hemodynamic instability, robotic adrenalectomy, pheochromocytoma, α-blockers, adrenal mass

## Abstract

**Background/Objectives:** Adrenalectomy for pheochromocytoma presents a risk due to catecholamine discharge, leading to perioperative hemodynamic instability and potential fatality. Recommendations stress surgical caution and care in referral centers. Laparoscopic and robotic adrenalectomy advancements have decreased perioperative risks, with robotic access deemed advantageous for larger tumors. This study aimed to assess if surgical technique and a new clevidipine-based perioperative protocol could improve hemodynamic stability. **Methods:** All robotic adrenalectomies treated in recent years (50) were included (Group A). A control group of 50 laparoscopic adrenalectomies (Group B) was also included. **Results:** In Group A, 7 patients had a BMI > 30 (14%), and 20 patients (40%) had pheochromocytomas > 5 cm in size. During surgery, 22 patients (44%) had systolic blood pressure/SBP ≥ 160 mmHg, and 18 patients (36%) had heart rate/HR ≥ 110 bpm. A total of 44 patients (88%) were treated perioperatively with clevidipine, 32 (64%) required amines perioperatively, and 8 (16%) did not require transfer to intensive care. In Group B, 2 patients had BMI > 30 (4%), 12 (24%) had pheochromocytomas > 5 cm in size, 35 (70%) had SBP ≥ 160 mmHg, 16 patients (32%) had HR ≥ 110 bpm, 23 (46%) patients required amines perioperatively, and all were transferred to intensive care after surgery. In addition, 11 (22%) patients were treated with clevidipine. In both groups, MAP < 60 mmHg was equivalent (62% vs. 60%). **Conclusions:** The clevidipine-based protocol, combined with robotic adrenalectomy, notably for larger tumors, potentially improves perioperative hemodynamic stability, reducing postoperative intensive care needs. This combination could represent an advancement in managing those patients.

## 1. Introduction

Since the 1950s, the mortality rate of pheochromocytoma has gradually decreased due to meticulous attention to perioperative blood pressure control and the optimization of volemia.

Preoperative medical preparation and treatment optimization are essential to reduce perioperative morbidity and mortality.

Optimal preoperative, perioperative and postoperative medical management is essential to reduce risks. In this perspective, the presence of a multidisciplinary team is important.

To date, there is no study defining the optimal treatment for the management of pheochromocytoma, but it is known that it should aim to optimize blood pressure and ensure intravascular euvolemia.

Maintaining good volemia allows for a reduction in perioperative hemodynamic fluctuations [1].

Medical preparation with α-adrenergic receptor blockers remains the mainstay for the prevention of life-threatening perioperative cardiovascular complications [2,3].

The introduction of α-blockers preoperatively has enabled the reduction in hemodynamic fluctuations during surgery [4].

However, despite good preoperative preparation, patients with pheochromocytoma may present with hemodynamic fluctuations during surgery that can sometimes be fatal.

Since 2013, although occasionally at first, we have introduced the use of clevidipine in the perioperative stabilization of patients undergoing adrenalectomy for pheochromocytoma.

This drug is a third-generation intravenous dihydropyridine calcium channel blocker.

The purpose of this article is to evaluate the use of this new molecule, clevidipine, on the hemodynamic stability of patients operated on for pheochromocytomas and, at the same time, to assess whether the surgical technique may have an influence on the results.

## 2. Materials and Methods

Our study is an observational retrospective study conducted in our tertiary referral Center of Endocrine Surgery in Marseille. We included only patients undergoing adrenalectomy for pheochromocytoma in 2 different periods according to the surgical technique performed (laparoscopic and robotic techniques) and the medical treatment used.

The rationale for choosing patients operated on in different time periods relies on the fact that until September 2021, all surgeries were performed only using a laparoscopic approach, and from this date onward, most were performed using a robotic technique.

Depending on the type of surgical technique, patients were divided into 2 groups: Group A and Group B. We excluded patients operated on via an open technique, those who underwent reinterventions for recurrence although they were small in number, and those with paragangliomas. We also excluded all patients with incomplete data and all patients who underwent adrenalectomy for another disease during the same period.

Preoperatively, all patients underwent comprehensive hormonal screening, morphologic and metabolic imaging, cardiologic evaluation, cardiac ultrasound, and anesthesiologic evaluation.

For all patients, the surgical indication was established after discussion by a multidisciplinary team. In the immediate postoperative period, patients were transferred to an intensive care unit depending on perioperative parameters, their hemodynamic instability, and the need to maintain intravenous amines postoperatively. This was an anesthesiologist’s decision.

For all included patients, we analyzed the following parameters:

- Age;
- Sex;
- BMI;
- Type of surgery: robotic adrenalectomy (Group A) and laparoscopic adrenalectomy (Group B);
- Size of adrenal mass;
- Systolic blood pressure (SBP) ≥ 160 mmHg during surgery;
- At least one episode of mean arterial pressure (MAP) < 60 mmHg during surgery;
- Heart rate (HR) ≥ 110 beats per minute (bpm) during surgery;
- The perioperative use of clevidipine;
- The perioperative use of amines;
- Transfer to an intensive care unit.

The literature defines the hemodynamic instability of SBP and HR to be above 30% of the normal range [5]; according to our previous study [6] and the five definitions already published about hemodynamic instability [5,6,7,8], we decided to analyze SBP ≥ 160 mmHg, at least one episode of MAP < 60 mmHg, and HR ≥ 110 bpm. The use of clevidipine was developed by our anesthesiology team after years of observation and follow-up of patients with pheochromocytoma. It was first used in 2013, but it was used sporadically for a while until recently; the clevidipine protocol is now used systematically.

## 3. Statistical Analysis

Comparison between the two groups was performed using the Pearson Chi-square test with IBM SPSS Statistics version 20.

We subsequently performed multiple correspondence analysis (MCA) with the R software version 4.4.1 with FactoMiner and factoextra packages.

## 4. Results

In our analysis, we included 100 patients undergoing adrenalectomy for pheochromocytoma between 2013 and 2023.

In detail, we included 50 consecutive patients undergoing robotic adrenalectomy between December 2021 (from this date onward, all adrenalectomies were performed by robotic approach) and September 2023, and we compared them with 50 laparoscopic adrenalectomies performed between February 2013 and December 2017.

We preferred to include these laparoscopic adrenalectomies because these were performed long before when the perioperative antihypertensive protocol was different and clevidipine was not used or used occasionally.

For 2013, we only included two patients as they were the first patients to be treated with clevidipine.

Surgery was performed by three experienced surgeons, and in both approaches, the technique was always transperitoneal adrenalectomy.

We divided the patients into two groups, each with 50 patients according to the surgical technique used:

- Robotic adrenalectomies: Group A.
- Laparoscopic adrenalectomies: Group B.

Of all the patients, 78 were women and 22 men.

The average age was 51 and the average BMI was 24.11.

The mean age was homogenous in both groups (51.6 in Group A and 50.5 in Group B).

In total, 33 patients had an adrenal mass ≥ 5 cm in diameter, and of these, only 12 (37.5%) had been operated on using a laparoscopic approach; the rest (62.5%) were treated using a robotic technique.

We analyzed the preoperative data below.

In Group A, 31 (62%) patients had preoperative hypertension, and all had antihypertensive therapy consisting of angiotensin II receptor blockers, angiotensin-converting enzyme inhibitors, beta-blockers, and calcium channel blockers. Of these, nine patients had two drugs, eight patients had three drugs, one patient had six drugs and the rest had monotherapy. Twenty (40%) patients had SBP > 130 mmHg 24–48 h before surgery. For the others, the pressure was between 90 and 130 mmHg. Thirty-nine (78%) patients had an HR between 72 and 102 bpm while for the rest of the patients, it was between 56 and 70 bpm. The average highest blood normetanephrine rate was 7.4 nmol/L (normal value ≤ 0.68 nmol/L) and the average highest blood metanephrine rate was 2.17 nmol/L (normal value ≤ 0.250 nmol/L).

In Group B, 32 (64%) patients had preoperative hypertension, and of these, only 23 (46%) had antihypertensive therapy. Of these, 10 (20%) patients had two drugs, and 3 (6%) patients had three drugs in combination. The rest of the patients only underwent monotherapy. Sixteen (32%) patients had SBP > 130 mmHg 24–48 h before surgery. For the others, the pressure was between 102 and 130 mmHg. Thirty (60%) patients had an HR between 72 and 110 bpm while for the rest of the patients, it was between 49 and 70 bpm.

The average highest blood normetanephrine rate was 9.6 nmol/L (normal value ≤ 0.68 nmol/L) and the average highest blood metanephrine rate was 0.75 nmol/L (normal value ≤ 0.250 nmol/L) (Table 1).

We report below the analysis performed in detail for each group.

In Group A, 7 patients had BMI > 30 (14%) and 20 patients (40%) had pheochromocytomas ≥ 5 cm in size.

In total, 22 patients (44%) had SBP ≥ 160 mmHg, 18 patients (36%) had HR ≥ 110 bpm, 31 patients (62%) had MAP < 60 mmHg, 44 patients (88%) were treated perioperatively with clevidipine, 32 (64%) required amines perioperatively, and 8 (16%) did not require transfer to intensive care (Table 2).

In Group B, 2 patients had BMI > 30 (4%), 12 (24%) had pheochromocytomas ≥ 5 cm in size, 35 (70%) had SBP ≥ 160 mmHg, and 16 patients (32%) had HR ≥ 110 bpm.

A total of 30 patients (60%) had MAP < 60 mmHg.

In addition, 23 (46%) patients required amines perioperatively. These patients in the intraoperative period had been treated with a combination of nicardipine hydrochloride (calcium channel blockers), esmolol hydrochloride (selective β-blocker), and clevidipine afterwards with vasoactive amines. Specifically, we noted that 13 patients treated with nicardipine hydrochloride and 8 treated with clevidipine required the administration of amines.

In total, 11 (22%) patients were treated with clevidipine in this group.

All patients in this group were transferred to intensive care after surgery (Table 2).

From the analysis by Pearson’s Chi-square test, we found a highly significant difference between the two groups regarding the following parameters:

- SBP ≥ 160 mmHg: *p* = 0.009 (Table 3).
- Perioperative use of clevidipine: *p* < 0.00001 (Table 4).
- Transfer to intensive care unit: *p* < 0.00001 (Table 5).

**Figure 1:**
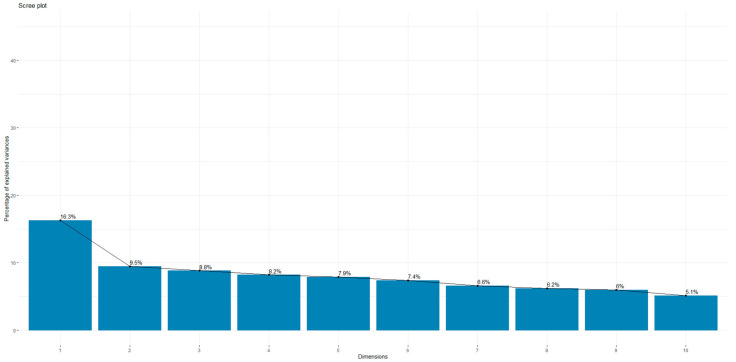
Percentage of explained variances in the overall dimensions.

**Figure 2:**
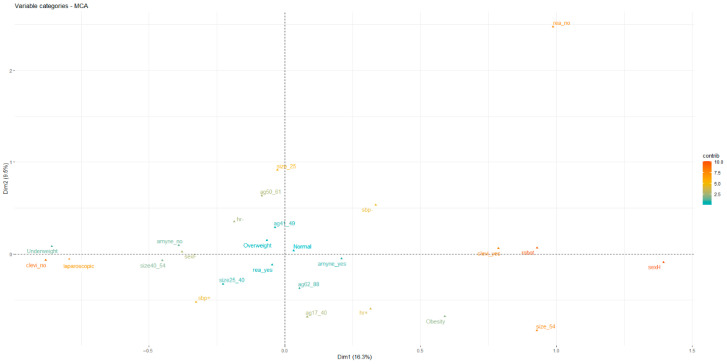
Multiple correspondence analysis (MCA) with X-axis (DIM1) and Y-axis (DIM2).

All variables considered were also analyzed using multiple correspondence analysis (MCA). The MCA (Figure 1 and Figure 2) for the robot group determined that obese men had adrenal masses > 54 mm, a smaller number of patients presented with SBP > 160 mmHg, none needed transfer to intensive care, and most had been treated with clevidipine.

The positive and negative X-axis represent population variables, including “type of surgery” combined with the perioperative use of “clevidipine”.

The positive and negative Y-axis represents “size of adrenal mass” and “transfer to intensive care unit”.

## 5. Discussion

Preoperative pheochromocytoma preparation methods and intraoperative treatment protocol are sources of continuous debate. For a very long time, α-blockers have been used in the stabilization of patients.

Studies carried out after the first introduction of α-blockers showed that patients who did not receive α-blockers had a higher rate of complications than patients treated with α-blockers (69% vs. 3%) [9,10].

Since the 1980s, α-blockade has been currently used in the management of pheochromocytoma, and the Roizen criteria are used to evaluate the efficacy of the treatment itself [11].

Systematic use of selective or nonselective α-blockers before surgery is strongly recommended even in normotensive patients.

It has been shown that this treatment is able to reduce perioperative mortality from 20–45% to 1–3%. However, all data available in the literature are based on observations, retrospective studies, and expert consensus, and the literature lacks randomized trials [4].

Among nonselective α-blockers, phenoxybenzamine is the molecule most commonly used as the first choice. It is an irreversible α1 and α2 blocker with a long duration of action (half-life of around 24 h). Over the past decade, selective α-blockade has begun to be abandoned in the United States in favor of selective α-blockers and calcium antagonists.

Doxazosin, prazosin, and terazosin are therefore alternatives, but they have hypotension and a short duration of action as undesirable effects [4].

Selective α-blockade has been described as inferior to phenoxybenzamine in preventing perioperative SBP fluctuations, despite the comparable risk of perioperative morbidity [12].

Despite optimal α-blocking, perioperative hemodynamic fluctuations may still be frequent but rarely associated with major cardiovascular events [4].

Normotensive patients present the same risks as hypertensive patients. They should therefore receive the same preparation, as there is an increased risk of hemodynamic instability, even though side effects appear to be greater in normotensive patients [3,13].

This point questioned the systematic use of α-blockers.

A previous study by our team found, in a large cohort of patients, no mortality and a low rate of global and cardiovascular morbidity, demonstrating that pheochromocytoma surgery without systematic preoperative pharmacological preparation and even without preoperative antihypertensives, for selected patients, is feasible and safe. This study also emphasized the importance of good preoperative patient and tumor assessment in order to optimize treatments and help identify high-risk patients, thus enabling better prevention and anticipation of possible complications [6].

In our experiment, we developed a protocol as described below.

Firstly, we recommend hydration and a high-salt diet before surgery.

Patients are admitted 48 h before surgery and receive an infusion of saline solution for 24 h before surgery.

We believe that intravascular volume is a problem that is often underestimated.

Volume expansion prevents severe hypotension and hypoperfusion in aggressive preoperative α-blockade [6]. We also believe that a personalized medical approach to preoperative drug treatment should always be proposed.

All of our patients therefore have a cardiologic and vascular evaluation in a specific department (dedicated to management of HBP) with preoperative ejection fraction assessment and are often admitted to the cardiology department before surgery where a customized antihypertensive treatment is developed and where they are monitored.

All patients with pheochromocytoma can benefit from a preoperative cardiological evaluation to determine the need for preoperative treatment, in particular, using α-blockers or calcium channel blockers.

Systematic administration of α-blockers prior to surgery should be discussed on a case-by-case basis by a multidisciplinary team.

Also, a list of contraindicated drugs should be systematically provided to the patient [14].

After consultation with our team of anesthesiologists, and after a long experience with adrenal surgery and based on the analysis of hemodynamic instability parameters, we started using clevidipine in 2013 to control hemodynamic fluctuations during adrenalectomy.

This was initially a sporadic use and often in combination with other calcium antagonists such as nicardipine hydrochloride and selective β-blockers such as esmolol hydrochloride.

The use of clevidipine in recent years, however, has become systematic since it has enabled better control of patients’ hemodynamic instability.

Since using clevidipine, we have found that patients are more stable even in the postoperative period so much so that patients often do not need to be transferred to intensive care (16% of patients did not require transfer in this analysis and more and more over the last year).

In this study, patients in Group A who were treated with the clevidipine protocol appeared to be more stable in the perioperative phase.

However, these same patients had been operated on using a robotic technique.

We questioned also whether the surgical technique could have an effect on the hemodynamic stability of patients.

Experiences reported in the literature are in favor of robotic surgery in reducing the effects of surgery on hemodynamic instability. In a study [15] that examined hemodynamic instability during surgery for pheochromocytoma and compared 101 retroperitoneal adrenalectomies and 240 transperitoneal adrenalectomies, the authors found that a retroperitoneal approach carries a higher risk of hypotension (MAP < 60 mmHg) than a transperitoneal one but they were comparable overall and for cardiovascular morbidity rates.

A recent systematic review and meta-analysis [16] that included 2985 patients concluded that the robotic technique is superior to the laparoscopy approach in managing adrenal tumors, even in the case of a specific adrenal tumor such as in pheochromocytoma.

The review and meta-analysis of Urabe et al. [17] showed that tumor size (six studies analyzed) and BMI (two studies included) were significantly related to hemodynamic instability.

Regarding the surgical technique, they found that open surgery and a retroperitoneal approach were associated with perioperative hemodynamic instability.

Other studies [18] point out that larger pheochromocytomas are more at risk of leading to hemodynamic instability. Voluminous masses have a more developed network of vessels and secrete higher levels of catecholamines that can lead to greater pressure fluctuations during surgery.

Natkaniec et al. [19] reported a significantly greater blood loss for pheochromocytomas larger than 6 cm in diameter than for those smaller in size.

In our analysis, in the group treated with a robotic approach and the clevidipine protocol (Group A), we found more voluminous adrenal masses compared with Group B (40% vs. 24% of adrenal mass ≥ 5 cm in size), and 16% of patients were transferred to the intensive care unit in contrast to Group B in which all patients were transferred postoperatively.

In addition, more patients with BMI > 30 are found in this group (14% vs. 4%).

Regarding hemodynamic fluctuations, Group A patients appear to be more stable: SBP ≥ 160 mmHg was found in 42% (70% in Group B). MCA, in Group A, showed that obese patients had an adrenal mass >54 mm in size, none were transferred to intensive care, and only a small percentage of patients had SBP ≥ 160 mmHg during surgery.

In our experience, the robotic technique allowed less manipulation of the adrenal masses and probably controlled the adrenergic discharge during surgery. This could also explain the greater stability of patients in the perioperative period as demonstrated by MCA. The statistical analysis employed (MCA) [20], by its qualitative nature, showed that both the use of clevidipine and the robotic technique are inversely related factors to transfer to intensive care. In fact, the graph regarding the statistical analysis performed in the same quadrant (X+ and Y+) corresponds to no transfer to intensive care. Both variables, therefore, appear decisive in preventing this undesirable event independently of each other.

However, we found no major differences in the two groups with regard to HR (HR ≥ 110 bpm 36% vs. 32%). We found no differences related to MAP > 60 mmHg (62% vs. 60%).

In this study, we compared two groups that are not homogeneous in terms of the type of medical treatment performed during surgery and we did not analyze preoperative antihypertensive treatment.

In Group B, only 11 patients (22%) were treated with clevidipine.

In Group A, we found a higher number of patients treated after pheochromocytoma removal with amines (64% vs. 46%).

These preliminary data show that Group A, treated with the new protocol and robotic technique, has better results in terms of hemodynamic stability, although it is not clear why most patients needed amine treatment. One explanation could be preoperative antihypertensive therapy, which we did not consider in this study, but undoubtedly, this finding needs further investigation.

There is no doubt that the use of the robotic platform entails higher costs. However, we believe that the higher costs due to the use of the robotic system could be balanced by reduced hospital stay and improved postoperative outcomes especially in more complex patients [21,22].

To achieve good results, a customized protocol should be used for each patient as described in the literature [23].

Recent publications comparing robotic and laparoscopic adrenalectomy have emphasized the advantages of the robotic technique for large adrenal masses in patients with BMI>30, including a reduction in operative time, length of hospital stay, and conversion rate to the open technique. The features of robotics, including 3D magnified stereoscopic vision, the greater degree of freedom compared to laparoscopy using multi-articulated robotic arms, the ability of the instruments to faithfully reproduce all the movements of the surgeon’s hand, and stable optics, may allow for limited manipulation of the mass by performing more delicate movements [24,25].

These results need further study, and we will be sure to perform further analysis in the future that can bring in more elements.

Our analysis has led to some interesting results. However, it should be considered that there are also some limitations. First, patients were included retrospectively so we could not include homogeneous groups of patients. In addition, our study is observational and preliminary and therefore needs further investigation. It is certain that patients with pheochromocytoma need adequate preparation in order to reduce risks during surgery. The reported mortality rate at anesthesia induction in cases of preoperatively undiagnosed and untreated pheochromocytoma is estimated at 80% [1,11].

The same insufflation and subsequent manipulation of the tumor can trigger a discharge of catecholamines capable of inducing severe hypertension and tachycardia, myocardial ischemia, or cerebral vascular accidents.

On the contrary, after ligation and sectioning of the main adrenal vein and progressive arterial devascularization of the pheochromocytoma, it is common to see a drop in tension that necessitates the administration of vasoconstrictive amines.

Therefore, close communication between the surgeon and anesthetist is of paramount importance to enable the administration of a large volume of bolus fluid just before vessel ligation and tumor devascularization.

Massive “fluid resuscitation” appears to be more effective than vasopressor administration.

It is not uncommon for the anesthetist to administer 2 to 3 L of fluid prior to vein ligation, in addition to rapidly stopping all vasodilators [1,6].

## 6. Conclusions

The challenge of the perioperative hemodynamic stabilization of patients with pheochromocytoma has always been debated. Numerous drugs are suggested for pre- and perioperative treatment. Surgical techniques over the years have also been considered. In our analysis, we wanted to analyze the role of surgical technique (robotic and laparoscopic) and a new perioperative protocol with clevidipine developed by our center and supported by a multidisciplinary team. We believe that the results obtained are promising. In the robotic group (Group A) treated with a new management approach, we found more voluminous adrenal masses (40% vs. 24%) and more obese patients (14% vs. 4%). In addition, patients appeared to be more stable in the perioperative phase (only 16% of patients were transferred to an intensive care unit). These results were very satisfactory. However, this study has the limitation of being a preliminary, retrospective, and observational study and needs further investigation.

## Figures and Tables

**Table 1:** Demographic characteristics. Normetanephrines: normal value = <0.68 nmol/L; metanephrines: normal value ≤ 0.250 nmol/L.

	Group A Robotic	Group B Laparoscopic
Gender		
● Man	22	0
● Woman	28	50
Average age	51.6	50.5
Size ≥ 5 cm	20 (40%)	12 (24%)
Preoperative hypertension	31 (62%)	32 (64%)
Preoperative SBP > 130 mmHg	20 (40%)	16 (32%)
Average highest blood normetanephrine rate	7.4 nmol/L	9.6 nmol/L
Average highest blood metanephrine rate	2.17 nmol/L	0.75

**Table 2:** Numbers of patients of the two groups, as well as percentages and *p*-values.

Surgical Technique	Group A Robotic	Group B Laparoscopic	*p*-Value
Size ≥ 5 cm	20 (40%)	12 (24%)	0.086
BMI ≥ 30	7 (14%)	2 (4%)	0.160
SBP ≥ 160 mmHg	22 (44%)	35 (70%)	0.009
HR > 110	18 (36%)	16 (32%)	0.673
MAP < 60 mmHg	31 (62%)	30 (60%)	0.838
Cleviprex	44 (88%)	11 (22%)	<0.001
Amines	32 (64%)	23 (46%)	0.070
No transfer to intensive care	8 (16%)	50 (100%)	<0.001

BMI: body mass index; SBP: systolic blood pressure; HR: heart rate; MAP: mean arterial pressure.

**Table 3:** A 2 × 2 contingency table comparing SBP ≥ 160 in the 2 groups. Significant results of Pearson Chi-square test: *p*-value 0.009.

SBP ≥ 160 mmHg	Group A	Group B	Total
yes	22	35	57
no	28	15	43
total	50	50	100

**Table 4:** A 2 × 2 contingency table comparing Cleviprex in the 2 groups. Significant results of Pearson Chi-square test: *p*-value 0.00001.

Cleviprex	Group A	Group B	Total
yes	44	11	55
no	6	39	45
total	50	50	100

**Table 5:** A 2 × 2 contingency table comparing transfer to intensive care unit in the 2 groups. Significant results of Pearson Chi-square test: *p*-value is 0.00001.

Transfer to Intensive Care Unit	Group A	Group B	Total
yes	8	50	58
no	42	0	42
total	50	50	100

## Data Availability

All data are available upon request.
